# Optimized Lysis-Extraction Method Combined With *IS6110*-Amplification for Detection of *Mycobacterium tuberculosis* in Paucibacillary Sputum Specimens

**DOI:** 10.3389/fmicb.2018.02224

**Published:** 2018-09-25

**Authors:** Pratt Kolia-Diafouka, Sylvain Godreuil, Arnaud Bourdin, Severine Carrère-Kremer, Laurent Kremer, Philippe Van de Perre, Edouard Tuaillon

**Affiliations:** ^1^Pathogenesis and Control of Chronic Infections, INSERM, EFS, Université de Montpellier, Montpellier, France; ^2^UMR MIVEGEC IRD-CNRS-Université de Montpellier, Montpellier, France; ^3^PhyMedExp, INSERM U1046, CNRS UMR 9214, Centre Hospitalier Régional Universitaire de Montpellier, Université de Montpellier, Montpellier, France; ^4^Centre National de la Recherche Scientifique, UMR 9004, Institut de Recherche en Infectiologie de Montpellier, Montpellier, France

**Keywords:** DNA extraction, *Mycobacterium tuberculosis*, polymerase chain reaction, *IS6110*, sputum

## Abstract

**Background:** When available, nucleic acid tests (NATs) offer powerful tools to strengthen the potential of tuberculosis (TB) diagnosis assays. The sensitivity of molecular assays is critical for detection of *Mycobacterium tuberculosis* (MTB) in paucibacillary sputum.

**Materials and Methods:** The impact of targeting repetitive *IS6110* sequences on the PCR sensitivity was evaluated across *mycobacterium* strains and reference material. Six lysis-extraction protocols were compared. Next, 92 clinical sputum specimens including 62 culture-positive samples were tested and the results were compared to sputum-smear microscopy, culture, and Xpert MTB/RIF test. Finally, the capacity to detect low MTB DNA concentrations was assessed in 40 samples containing <1.5 × 10^2^ copies/ml *ex vivo* or after dilution.

**Results:** The lower limit of detection (LOD) using the *IS6110* PCR was 107 genome copies/ml (95% CI: 83–130) using MTB H37Rv as a reference strain, versus 741 genome copies/ml (95% CI: 575–1094) using the *senX3* PCR. The proportion of recovered MTB DNA after lysis and extraction ranged from 35 to 82%. The Chelex^®^ method appeared as a more efficient protocol among the six different protocols tested. The sensitivity and specificity in clinical sputum samples were 95.1% (95% CI: 90.7–99.6) and 100% (95% CI: 96.2–100.8), respectively. Among 40 samples with low MTB DNA concentration, 75% tested positive for *IS6110* PCR, versus 55% using the Xpert MTB/RIF assay (*p* = 0.03).

**Conclusion:** Laboratory assays based on an efficient MTB lysis and DNA extraction protocols combined with amplification of *IS6110* repeat sequences appear as a sensitive diagnostic method to detect MTB DNA in sputum with low bacterial load.

## Introduction

Tuberculosis (TB) is a deadliest infectious disease, accounting for about 10.4 million new cases and 1.3 million deaths worldwide in 2016 (World Health Organization, 2017). A major priority and a challenge for TB control are to strengthen the capacity to diagnose the disease. Mycobacterial culture remains the gold standard test for TB diagnosis in high resource settings. Culture has high sensitivity with a limit of detection (LOD) to 10–100 cfu/ml, but the time-to-result ranges from 2 to 8 weeks (American Thoracic Society, 2000) and this method requires a BSL-3 laboratory facility. In most low resource settings, bacterial culture, however, is unavailable, leaving sputum smear microscopy as the major direct bacteriological test for TB diagnosis (Wejse, 2014). However, the LOD of the unconcentrated smear test is approximately 10,000 acid-fast bacilli (AFB)/ml, and microscopy has suboptimal specificity partially due to the possible contamination by non-tuberculosis mycobacteria (American Thoracic Society, 2000).

Nucleic acid tests (NATs) are viewed as a potential mean of overcoming these barriers, and as a new standard practice for TB diagnosis (Huggett et al., 2009). Accurate and sensitive detection of *Mycobacterium tuberculosis* (MTB) DNA in clinical paucibacillary specimens hinges on combination of efficient lysis of the bacilli, DNA extraction, removal of PCR inhibitors, and amplification of low concentration of the target sequence. MTB cell wall is resistant to conventional bacterial lysis techniques due to the complex structure of lipophilic molecules, including the long-chain mycolic acids (Brennan and Nikaido, 1995) and polysaccharides. Sputum samples contain PCR inhibitors that also contribute to make challenging DNA extraction and amplification. A wide variety of sputum processing protocols has been described using sonication, boiling, SDS treatment with lysozyme and heating, or exposure to proteinase K or chaotropic salts (Garg et al., 2003). Different approaches have been also used for NAT-based on amplification of single or repeated genomic PCR targets, such as *IS6110, rpoB* ( Meghdadi et al., 2015), and other. However, the *IS6110* sequence remains probably the most frequently used and extensively studied PCR target. *IS6110* is a coding transposase sequence present in a multiple copies number depending on the strain, ranging from 1 to 20 with a mean of 10 copies per bacilli (Gutierrez et al., 1998) and is only found in members of the MTB complex (Thierry et al., 1990). Previous studies have reported higher sensitivities of PCR methods based on amplification of the *IS6110* multi-copy element when compared to methods relying on single copy genes (Luo et al., 2010). Cepheid has recently launched a new GeneXpert^®^ cartridge including *IS6110* and *IS1081* amplification to improve the detection rate of smear-negative TB. Interestingly, increased clinical sensitivity was reported with this new assay, particularly in children and in HIV co-infected individuals, representing populations often refractory to TB diagnosis, compared to the *rpoB* based cartridge (Dorman et al., 2018).

A systematic approach evaluating each step of MTB molecular assays is requested to better understand the determinants of the assay performance on low bacterial load specimens. In this study, we first developed and assessed in details in-house *IS6110* assay versus single-copy gene amplification. Second, we evaluated different lysis-extraction protocols to determine the most efficient methods. Finally, we assessed the performance of the optimized molecular assay on clinical samples and compared the results to sputum-smear microscopy, culture, and a commercial automated PCR.

## Materials and Methods

### Bacterial Strains, Genomic DNA, and Clinical Samples

*Mycobacterium tuberculosis* mc^2^7000, a derivative of *M. tuberculosis* H37Rv (Sambandamurthy et al., 2006) was kindly provided by William Jacobs (Albert Einstein College of Medicine, Bronx, NY, United States). The *Mycobacterium bovis* BCG Pasteur strain contains a single copy of *IS6110* whereas *M. tuberculosis* mc^2^7000 contains 16 reiterations of this sequence (Alonso et al., 2013). Both *M. tuberculosis* mc^2^7000 and *M. bovis* BCG were grown in Middlebrook 7H9 medium (Difco, Detroit, MI, United States) containing 100 μg/ml pantothenic supplemented with 0.05% Tween80 (Sigma-Aldrich, St. Louis, MO, United States), 0.02% glycerol (Sigma-Aldrich, St. Louis, MO, United States) and 10% oleic acid, dextrose, catalase (OADC) enrichment (Becton Dickinson, Baltimore, MD, United States) and Sauton’s medium, respectively, at 37°C for 2–3 weeks in BSL3 laboratory. Genomic DNA of *M. tuberculosis mc^2^7000* and *M. bovis BCG* were extracted using Cetyltrimethylammonium bromide (CTAB) extraction as previously described (Larsen et al., 2007), and used for analytical performance of *IS6110* and *senX3* PCRs assay. Cultures of *M. tuberculosis mc^2^7000* were quantified by plating on Middlebrook 7H10 agar (Difco, Detroit, MI, United States), and aliquots of approximately 2 × 10^8^ cfu/ml were stored at -80°C for later use.

The efficiency of six MTB DNA extraction methods was performed in phosphate-buffered saline (PBS) and in spiked sputum. The discarded excess sputum originally submitted for routine Gram staining and bacterial culture, were spiked with diluted cultures of bacterial cfu. The previous quantified aliquots of *M. tuberculosis mc^2^7000* have been thawed and suspended in (PBS) with 0.05% Tween80 (Sigma-Aldrich, St. Louis, MO, United States), then forced through a 21-gauge needle with a syringe to break up cell clumps (Stokes et al., 2004). The cells were seven 10-fold diluted in TE buffer (10 mM Tris, 1 mM EDTA, pH 8.4) and vortexed for 1 min to disrupt any residual clumps.

Two hundred microliters of each appropriate concentration of bacilli, diluted in TE buffer, was added to 1.8 ml of MTB-negative sputum to prepare the spiked sputum samples. The spiked sputum were treated according to the normal sample-processing protocol as if it had come from a patient suspected of having TB. The assay used 10 replicates per dilution for extraction methods, and testing of each dilution was repeated eight times.

Ninety-two sputum samples (62 TB positive and 30 negative control) were collected consecutively at the Montpellier University Hospital (NCT number: NCT02898623) and stored at -20°C until used for the *IS6110* PCR evaluation. Digested and decontaminated sputum samples with MycoPrep^®^ kit (Becton Dickinson, Baltimore, MD, United States), were cultured in both BACTEC^TM^ MGIT^TM^ 960 Mycobacterial Detection System (Becton Dickinson Microbiology Systems, Sparks, NV, United States) and Löwenstein–Jensen medium (BioMérieux, Marcy l’Etoile, France). TB culture was considered as the gold standard to evaluate the clinical performances of molecular assays. Sixty-two specimens were TB culture-positive and 30 were TB culture negative, and used as negative control. Among 62 TB culture-positive, 50 were tested positive by both smear and culture and 12 were culture-positive but smear-negative. Smear grading was determined (Technical Guide, 2000). All smear negative samples and samples with *M. tuberculosis* DNA level below the LOD of smear test were defined as paucibacillary specimens [<10,000 acid-fast bacilli (AFB)/ml].

### Methods of Extracting MTBC DNA From Culture in PBS and in Sputum

For all six extractions methods, each of which was repeated eight times, the spiked respiratory specimens were centrifuged at 3000 × *g* for 20 min. The supernatants were discarded, and pellets were processed for each extraction methods as follow: (i) Chelex^®^ method: incubation with 200 μl of 20% Chelex^®^ 100 resin (Bio-Rad, Richmond, CA, United States) prepared in TE buffer [10 mM Tris–HCl (pH 8.0), 1 mM EDTA] (Sigma-Aldrich, Germany). After vortex mixing, boiling at 100°C for 15 min, then placed in an ultrasonic water bath for 15 min. After centrifugation at 14,000 *g* for 5 min, the supernatant was used for qPCR. (ii) Guanidium Isothicyanate (GTIC) method: incubation with 200 μl of lysis buffer (10 mM Tris–HCl, 1 mM EDTA, 1 M GTIC, 0.5 M NaCl) for 20 min, combined with 3 cycles of freeze-thawing (-80°C for 5 min and 100°C for 5 min) and boiling at 100°C for 15 min. (iii) Tween 20 method: suspension in 200 μl of lysis buffer [0.45% Tween 20, 50 mM Tris–HCl (pH 8.0), 50 mM KCl, 2.5 mM MgCl_2_], containing 70 μl of 10 mg/ml Lysozyme and was incubated at 37°C for 1 h. 30 μl of proteinase K (10 mg/ml, Qiagen, Germany) and 2% SDS were added, followed by incubation for 1 h at 56°C to remove PCR inhibitors and heated for 15 min at 100°C to ensure complete mycobacterial lysis. (iv) Non-idet P-40 method: the cell pellet was suspended and subjected to method (iii) but NP-40 instead of Tween 20. (v) Triton method: incubation with 200 μl of lysis buffer [100 mM NaCl, 10 mM Tris–HCl (pH 8.0); 1 mM EDTA and 1% Triton X-100]; was incubated for 20 min at 95°C. (vi) NaOH method: incubation with 200 μl lysis buffer [10 mM Tris–HCl (pH 8.0); 1 mM EDTA, 50 mM sodium hydroxide (NaOH) and 2% SDS] at 95°C for 5 min. A total 1800 μl of pure water was added, then vortex mixed, followed by boiling for 15 min at 100°C. After vortex mixing, the tubes were placed in ultrasonic bath for 15 min. For methods (ii) to (vi), after centrifugation at 14,000 *g* for 5 min, the supernatant was transferred to a new tube. Then supernatant was purified using traditional nucleic acid precipitation: precipitate DNA with 2 volume of ice-cold ethanol and 1/10th volume 3 M sodium acetate, kept at -20°C for 20 min. After centrifugation tubes at 14,000 *g* for 10 min at 4°C, the pellet was washed with 70% ethanol, air dried, then resuspended in 50 μl TE buffer and used 5 μl in PCR. Chelex^®^ resin methods in act as chelating groups inactivating nucleases and protecting DNA by binding polyvalent metal ions such as magnesium (Mg^2+^). After boiling the resin and cell residues are pelleted, and the supernatant containing the DNA is removed. The pellet was eluted in 100 μl of TE, and used for PCR. The efficiency of cell lysis and DNA recovery for each method was assessed using the *IS6110* PCR assay, based on the proportion of DNA recovered relatively to the estimate input quantity of DNA The performance of the six DNA extractions methods were compared using mean differences in *C*_t_ values and the end point PCR for each extraction methods.

### Quantitative Real-Time PCR

All PCR was performed using LightCycler 480 Real-Time PCR System (Roche Applied Science, Germany). PCR was performed in 20 μl final reaction volume, containing 5 μl of DNA, and 5× DNA polymerase mix (Omunis, Clapiers, France). The following thermal profile was used: 95°C for 15 min and amplification 95°C for 15 s following 60°C for 1 min during 50 cycles. A heterologous internal control (IC) using a Cy5 probe and having a target *C*τ value ranging from 32 to 34 was added to control DNA extraction and amplification (Omunis, Clapiers, France). The standard curve was calculated automatically by plotting the *C*τ values against four dilutions of the standard and by extrapolating the linear regression line of this curve.

**FIGURE 1:**
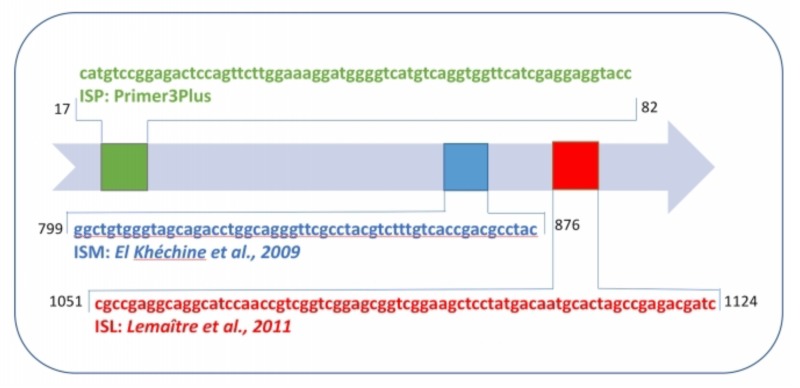
Schematic representation of *IS6110* sequence and primer-probe sets of ISP, ISM, and ISL.

Three PCR targeting *IS6110* elements were developed. Two *IS6110* PCRs were based on primers and probes previously reported (El Khéchine et al., 2009; Armand et al., 2011), in blue and red nucleic acid sequences (**Figure 1** and **Table 1**). A third set of primers and probe were designed, using the Primer3Plus software, within the *IS6110* targeted insertion (green nucleic acid sequence) based on the complete genome sequence of *M. tuberculosis* H37Rv (Genbank, number NC_000962.3). *M. tuberculosis* H37Rv international standard (Advanced Biotechnologies Inc., Eldersburg, MD, United States) was used to generate an accurate standard curve of PCR using concentrations. *senX3* PCR was performed, *from* other primers and probe designed to target the *senX3-regX3* intergenic region, as previously described (Queipo-Ortuño et al., 2009).

**Table 1:** Primer-probe sets used in this study.

Target	Primers/Probes	Amplicon size (bp)
***IS6110* (MULTICOPY)**		
ISL	CGCCGAGGCAGGCATCCAACC	73
	GATCGTCTCGGCTAGTGCATT	
	FAM-CGGTCGGAAGCTCCTATGAC-TAMRA	
ISM	GGCTGTGGGTAGCAGACCT	77
	GTAGGCGTCGGTGACAAAG FAM-	
	GGGCAGGGTTCGCCTACGT-TAMRA	
ISP	CATGTCCGGAGACTCCAGTT	66
	GGTACCTCCTCGATGAACCA FAM-	
	AAAGGATGGGGTCATGTCAG-TAMRA	
*senX3* (single-copy)	CGCGGCTAATCACGACGGCACG	164
	CTCTTCCTCTCGTTGTGACCTG	
	HEX-CCTATCACGACGACGAGCGACCCGA-BHQ-1	


The specificity of the primers was first verified by using NCBI BLAST algorithm, followed by real-time PCR specificity testing with DNA extracted from the reference strains. Genomic DNA from 6 mycobacterial species (*Mycobacterium fortuitum, Mycobacterium avium, Mycobacterium xenopi, Mycobacterium gordonae, Mycobacterium intracellulare,* and *Mycobacterium abscessus)* was used as the template for the specificity. *IS6110* probes incorporate a 5′ FAM reporter, whereas the *senX3* probe uses a 5′ VIC reporter.

### Analytical Performances of the PCR Assays on Genomic MTB DNA

Performances of the assays were assessed using genomic DNA from *M. tuberculosis* mc^2^7000 and *M. bovis* BCG, then were quantified using Qubit^®^ fluorescent dyes quantitation method (Thermo Fisher Scientific); and using clinical specimens. Direct smears were prepared from the specimens and stained using the Ziehl-Neelsen and auramine staining method. The linear dynamic range of the qPCR assays, variability inter and intra run were evaluated by plotting separately the results of 10 replicates of a 10-fold serial dilutions using the *M. tuberculosis* H37Rv commercial standard, *Mycobacterium bovis* BCG DNA and *M. tuberculosis mc^2^7000* DNA. The lower LOD (LOD) of the three qPCR targeting *IS6110*, of duplex and triplex combination of primer and probe set, were compared.

For comparisons, 40 sputum specimens culture positive were randomly chosen, and also tested for TB DNA using the Xpert MTB/RIF test a fully automated real-time PCR endorsed by WHO in 2010 for TB diagnosis and rifampicin resistance testing (Lawn et al., 2013). Thirty-two decontaminated and digested specimens diluted in PBS at the lower LOD level (LOD) of GeneXpert test, namely 131 cfu/ml (Helb et al., 2010), whereas eight sputum samples with TB DNA concentration below 1.5 × 10^2^ copies/ml were used without dilution, to evaluate the performance of the assays for low TB DNA sputum concentrations.

### Xpert Protocol

Xpert MTB/RIF was used according to manufacturer’s recommendations. Xpert MTB/RIF uses hemi nested real-time PCR assay to amplify the RNA polymerase β subunit gene (*rpoB*), which is explored with molecular beacon technology. The sample treatment and cartridge loading processes used were done according to the manufacturer’s instructions. Briefly, each diluted sediment of 500 μl was mixed with 1.5 ml of a commercial NaOH- and isopropanol-containing sample reagent (Sample Reagent; Cepheid, Sunnyvale, CA, United States). The mixture was incubated for 15 min at room temperature with vigorously shaking, and then added to the sample-loading chamber of the cartridge for automatic processing, and the result was available within 2 h.

### Statistics

Regression analysis between assigned and observed values was used for linearity assessment. The median values, interquartile ranges of each concentration and regression coefficient were determined. The probit method was used to determine the LOD. The LOD was read off the generated graph at the 95% probability for response. SPSS software (Statistical Package for Social Sciences; IBM, Chicago, IL, United States) was used for probit regression analysis. Bland–Altman bias plots were used to assess differences between repeated and single target PCR assays on MTB strains. For each plot, mean bias and 95% confidence interval of the bias were calculated and the mean biases were compared using Student’s *t*-test. Statistical analyses were done with MS Excel and GraphPad Prism 6.0 (GraphPad Software, Inc., San Diego, CA, United States).

## Results

### Analytical Sensitivity of PCR Assays Targeting *IS6110* Repeated Sequences Versus *senX3* Sequence

**FIGURE 2:**
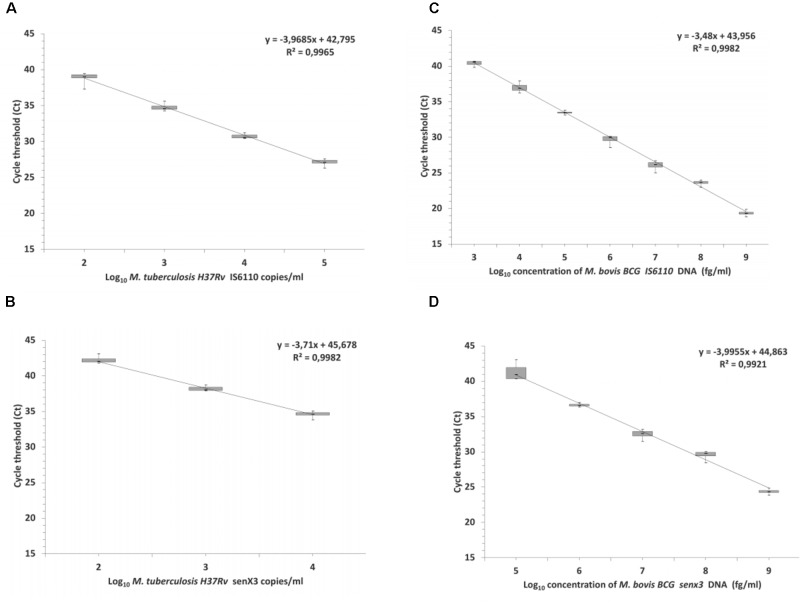
Linearity of *IS6110* (ISP) and *senX3* qPCR. *M. tuberculosis* H37Rv commercial standard **(A,B)** and *M. bovis* BCG genomic DNA **(C,D)**.

**FIGURE 3:**
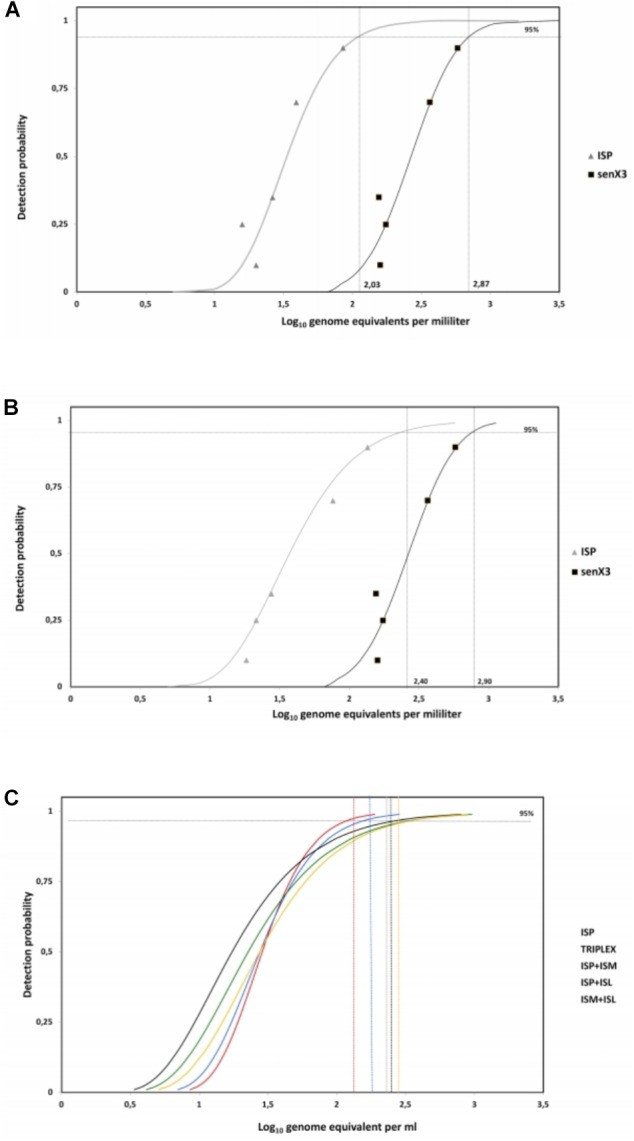
Detection limits of *IS6110* and *senX3* PCR assays. Curves determined by probit analysis (95% probability detection). **(A)** *IS6110* and *senX3* PCR using *M. tuberculosis* mc^2^7000 DNA. LOD were estimated at 2.03 log_10_ (107 copies/ml) and 2.89 log_10_ (741 copies/ml), respectively. **(B)** The detection limit of *M. bovis* BCG was 2.40 log_10_ and 2.90 log_10_ for *IS6110* and *senX3* PCR assays, respectively. **(C)** *IS6110* PCR detection limits using two or three sets of primers compared to a single set of primers based on *M. tuberculosis* mc^2^7000 strain were estimated at 2.1, 2.2, 2.3, 2.35, 2.4 log_10,_ respectively for ISP, Triplex, ISP+ISM, ISP+ISL, and ISM+ISL.

High degree of linearity of DNA measures using *IS6110* and *senX3* PCR assays were observed from 100 to 100,000 *M. tuberculosis* H37Rv copies/ml and 10 ng to 10 fg *M. bovis* BCG genomic DNA (**Figure 2**). The LOD for *M. tuberculosis* H37Rv was estimated to 107 (95% CI: 83–130 copies/ml) and 741 (95% CI: 575–1094 copies/ml) genome copies/ml using *IS6110* and *senX3* PCR, respectively (**Figure 2A**). For *M. bovis* BCG, the estimated LOD was 251 (95% CI: 101–500) and 794 genome copies/ml (95% CI: 628–964) for *IS6110* and *senX3* PCR, respectively (**Figures 3A,B**). The PCR was negative for all six mycobacteria species tested, confirming the specificity of the PCR assays.

The intra and inter-run variability was assessed by evaluating the standard deviation of threshold cycles (*C*_t_) in five independent PCR runs, of 10 replicate. Average and range were below 10% for all strains and PCR assays (**Table 2**).

**Table 2:** Intra run and inter run variability.

	In-house qPCR *IS6110*			In-house qPCR*senX3*		
		
	Mean (Log10 fg/μl)	*SD*	%CV	Mean	*SD*	%CV
**INTRA RUN MTB**						
6 log_ 10_	5.85	0.05	1.36	5.53	0.17	0.70
5 log_10_	5.07	0.09	2.03	4.73	0.11	0.45
4 log_10_	3.74	0.19	3.55	3.17	0.07	0.34
3 log_10_	2.53	0.16	2.27	2.59	0.13	2.29
2 log_10_	1.70	0.08	1.14	ND	ND	ND
1 log_10_	0.80	0.11	6.25	ND	ND	ND
**INTRA RUN *BCG***						
6 log_ 10_	5.55	0.52	3.26	5.70	0.51	2.61
5 log_10_	4.67	0.05	3.16	4.22	0.15	0.66
4 log_10_	3.40	0.22	2.56	3.15	0.41	1.41
3 log_10_	2.73	1.19	5.31	2.56	0.25	0.76
2 log_10_	1.86	0.10	2.53	1.53	0.82	2.27
1 log_10_	0.83	0.66	18.32	ND	ND	ND
**STANDARD**						
5 log_ 10_	4.99	0.12	0.46	4.89	0.05	4.14
4 log_10_	3.98	0.27	0.88	3.88	0.07	0.61
3 log_10_	3.07	0.10	0.30	3.00	0.00	0.84
2 log_10_	1.96	0.83	2.21	ND	ND	ND
Average Mtb	1.86	0.11	2.76	4.01	0.12	0.95
Average BCG	0.83	0.45	3.02	3.43	0.43	1.54
**INTER RUN**						
**Operator 1**						
5 log_10_	4.96	0.04	4.00	4.95	0.15	3.16
4 log_10_	3.99	0.14	0.60	4.01	0.19	4.60
3 log_10_	3.02	0.05	0.55	2.99	0.25	2.55
2 log_10_	1.94	0.09	6.23	1.98	0.11	2.70
**Operator 2**						
5 log_10_	4.97	0.02	6.15	4.93	0.05	3.15
4 log_10_	4.01	0.03	4.80	4.07	0.19	4.60
3 log_10_	2.99	0.44	1.55	2.96	0.64	2.55
2 log_10_	2.10	0.17	15.35	1.97	0.20	3.53
Average	3.52	0.17	6.96	3.98	0.25	2.96


Different format of duplex or triplex PCR, combining two or three sets of primers targeting *IS6110* were compared for their capacity to detect the low concentration of *M. tuberculosis* mc^2^7000 genome, but without significant difference in the LOD (*P* = 0.067) (**Figure 3C**).

### Impact of *IS6110* PCR Versus *senX3* Sequence PCR on MTB and *M. bovis* BCG on DNA Quantification

**FIGURE 4:**
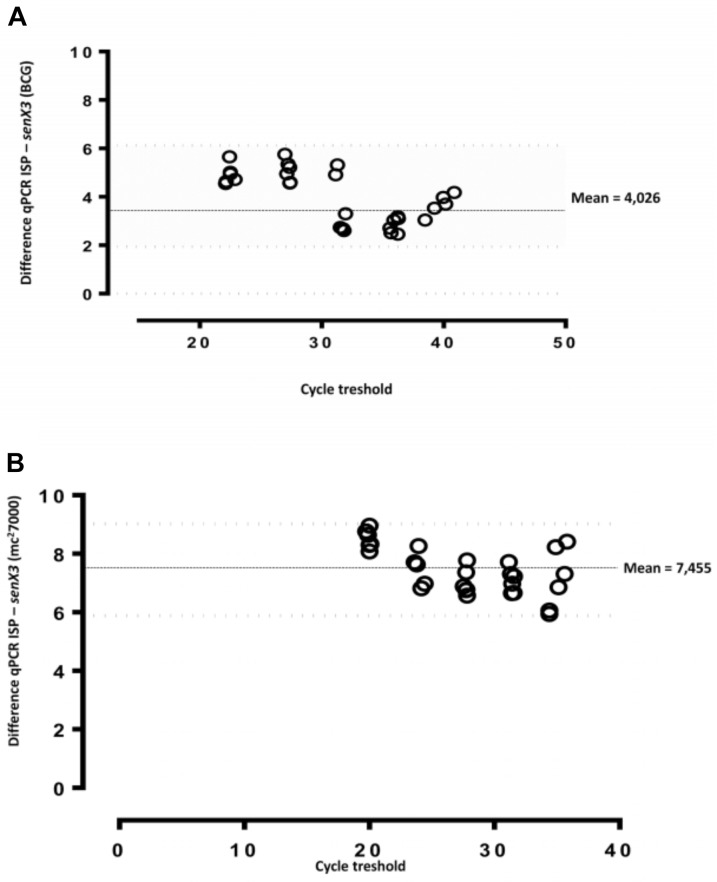
Bland–Altman bias plots for two different quantitative MTBC DNA real-time PCR assays. Five serial dilutions of *M. bovis* BCG **(A)** and *M. tuberculosis* mc^2^7000 **(B)** strain were tested for MTBC DNA quantification by *IS6110* and *senX3* PCR assays. The mean bias was determined to be 4.026 and 7.455-cycle threshold for *M. bovis* BCG and *M. tuberculosis* mc^2^7000, respectively.

Serial dilutions of genomic DNA isolated from *M. tuberculosis* mc^2^7000 or *M. bovis* BCG were tested with *IS6110* and *senX3* PCRs. Results were compared using Bland–Altman bias plots (**Figure 4**). The differences in DNA levels between *IS6110* versus *senX3* assays were 4.03 C_T_ (95% CI: 1.6–6.4) for *M. bovis* BCG (**Figure 3A**) and 7.45 C_T_ (95% CI: 5.9–9.0) for *M. tuberculosis* mc^2^7000 (**Figure 3B**), respectively.

### Comparison of the Efficiency of MTB DNA Extraction Protocols

**FIGURE 5:**
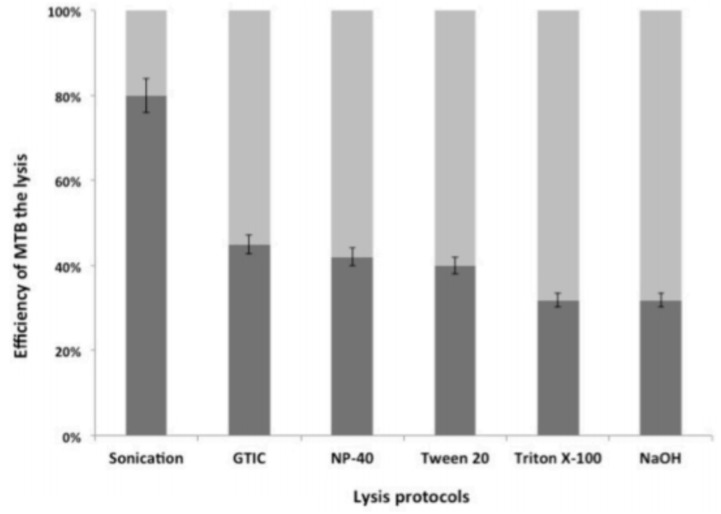
Efficacy of MTB lysis using six different lysis methods combined with the Chelex^®^ resin extraction. Each column represents average DNA copy number per microliter obtained in five independent experiment with three replicate reactions.

**FIGURE 6:**
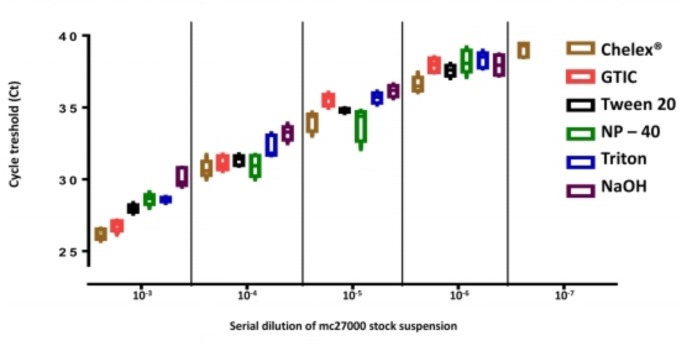
Comparison of DNA extraction protocols in spiked sputum samples. The *M. tuberculosis mc^2^7000* stock suspension was diluted and used to spike negative sputum samples. Box plots with C_T_ median, 10th, 25th, 75th, and 90th centiles of 10 replicates. Methods are indicated by colors: brown: Chelex^®^ method; pink: Guanidium Isothicyanate/Tris-HCl/EDTA + 3 cycles of freeze thawing and boiling; black: Tween 20/Tris-HCl/EDTA/lysozyme+proteinase K/SDS + warming cycles 56°C/95°C; green: Nonidet P-40/Tris-HCl/EDTA/lysozyme+proteinase K/SDS + warming cycles 56°C/95°C; blue: Triton X-100/Tris-HCl/EDTA; purple: NaOH + boiling and sonication.

The efficiency of cell disruption methods was compared by evaluating the quantities of MTB DNA recovered and quantified relatively to the theoretical input of *M. tuberculosis* mc^2^7000 strain (cfu/ml) by *IS6110* PCR. The proportion of recovered MTB DNA ranged from 35 to 82% (**Figure 5**). Next, seven dilutions (10^-1^ to 10^-7^) of *M. tuberculosis* mc^2^7000 strains in PBS -0.05% Tween 80; in experimentally contaminated sputum and PBS were tested. The Chelex^®^ method appeared as a more efficient protocol among the six methods tested. *C*_t_ values of the internal control were within the recommended range (32–34) in all samples. The lowest concentration detected by the *IS6110* PCR was at dilution 10^-6^ using the NaOH, Tween 20, Triton X-100, NP-40 protocol, and dilution at 10^-7^ using the Chelex^®^ method (*p* = 0.002; **Figure 6**). The efficiency of DNA extraction was comparable in spiked sputum and in PBS (data not shown).

### Performances of *IS6110* PCR Assays Using the Chelex^®^ Extraction Method

**FIGURE 7:**
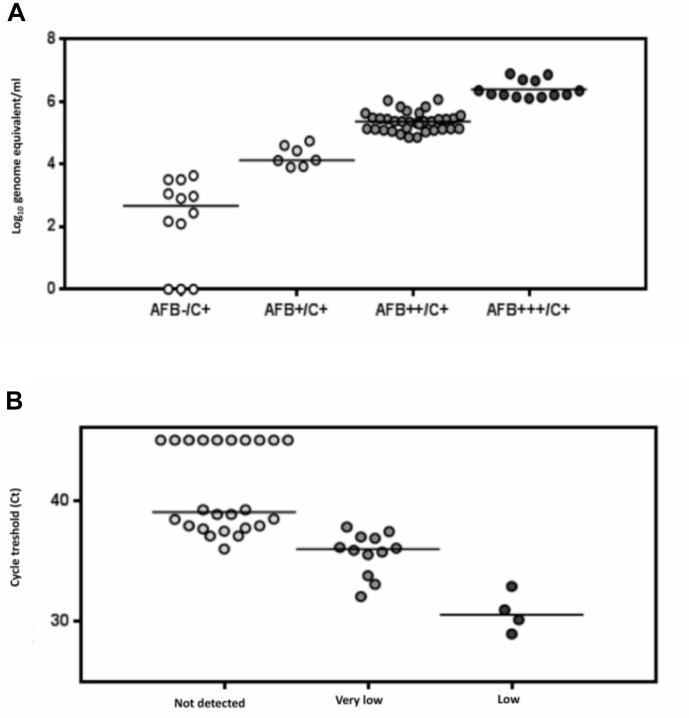
MTB DNA concentration in culture positive samples stratified according to smear microscopy results using the *IS6110* PCR **(A)**. Comparison of real-time PCR *IS6110* assay and Xpert MTB/RIF on 40 clinical sputum samples. Positive threshold of the PCR assay is indicated by the dotted line **(B)**.

All the sputum were tested by real-time *IS6110* PCR. The performance of Chelex^®^ method combined with *IS6110* PCR was evaluated on 62 sputum. All controls were tested negative for TB DNA. We observed an inversely proportional relationship between the *C*_t_ values and the number of AFB detected in culture positive samples (**Figure 7A**). The sensitivity, specificity, PPV and NPV of the *IS6110* in house real-time PCR in clinical samples were 95.1% (95% CI: 90.7–99.6), 100% (95% CI: 96.2–100), 100%, and 93.7% respectively. The sensitivity of the *IS6110* PCR was 100 and 75%, for smear-positive and smear-negative samples, respectively.

Forty specimens tested positive with the *IS6110* PCR, were randomly selected for comparison with the Xpert assay. Of the 40 specimens, 32 were serial diluted until a target concentration close the Xpert assay LOD (131 cfu/ml) (Helb et al., 2010), whereas eight sputum samples with TB DNA concentration below 1.5 × 10^2^ copies/ml were used without dilution. Samples tested more frequently positive using the *IS6110* PCR than using the Xpert assay (75 vs. 55%, *p* = 0.03), 20 specimens tested positive for TB DNA with the two PCR assays (50%), 10 were found positive only with the *IS6110* assay (25%), and 10 were found negative with the two PCR assays (25%). The median threshold cycle (*C*_t_) value for the *IS6110* PCR was 38.64 when sputum tested negative for TB DNA using the Xpert (**Figure 7B**).

## Discussion

The sensitivity of molecular assays is critical for MTB detection in low bacterial load specimens. In this study, we analyzed in a comprehensive way the different steps of PCR methods and identified the most efficient combination of MTB lysis, DNA extraction and amplification protocols to obtain a rapid and cost-effective MTB DNA detection. Testing clinical samples characterized by microscopic examination, culture and commercial NAT, confirmed the high sensitivity of the *IS6110* specific PCR when used in combination with a Chelex^®^ method.

The analytical sensitivity is an essential characteristic of molecular assays that should be constantly determined (Burd, 2010). Few studies have assessed the LOD of *M. tuberculosis* PCR (Barletta et al., 2014; Reed et al., 2016). In addition, the LOD were unfrequently tested by repeated measure in a narrow dilution ranges around the threshold value, as recommended (Tholen et al., 2003). Our study confirmed and determined accurately the gain in analytical sensitivity related to the target of the *IS6110* repeat sequences. A fourfold difference (0.38 log_10_ genome/ml difference) was observed in the LOD of the *IS6110* PCR assay testing MTB strain containing 16 sequences per genome versus one copy for *M. bovis* BCG. The LOD of the *IS6110* was estimated around 100 genome copies/ml using the *M. tuberculosis* mc^2^7000 strain, which is sevenfold lower (0.83 log_10_ genome/ml difference) to the *senX3* LOD, confirming the gain related to the target of the repeated sequence. The comparison of different *IS6110* PCR and different multiplex PCR combinations did not further improve the sensitivity. A gain in analytical sensitivity was expected since the number of primers and probes were multiplied (Armstrong et al., 2012). This result was somewhat disappointing but may be explained by the cluttering of primers and polymerase on the target sequence.

Besides nucleic acid amplification, DNA extraction is the other critical step for detection of low mycobacterium bacilli concentrations observed in sputum smear-negative specimens. Methods dedicated to mycobacterial DNA extraction has to fulfill four key objectives: (1) lysis of the thick and waxy cell wall, (2) removal of non-nucleic acid organic and inorganic molecules that may impair DNA amplification, (3) minimize the nucleic acid loss, and (4) keep DNA integrity throughout the extraction/purification process. We have compared six lysis and extraction protocols adapted from previous studies (Honore et al., 2001; Heginbothom et al., 2003; Honoré-Bouakline et al., 2003; Bahador et al., 2004; Leung et al., 2011) to select the best performing method. According to our results, the Chelex^®^ method provides the best efficiency for recovering *M. tuberculosis* DNA from sputum samples. Sputum specimens pose challenges for specific microbial detection because of the presence of endogenous PCR inhibitors and contaminating DNA from the normal flora (Amicosante et al., 1995; Böddinghaus et al., 2001). Interestingly, comparable MTB detection in both PBS and sputum samples confirmed the (near) absence of PCR inhibition using the Chelex^®^ method. Moreover, being rapid, without the requirement of detergents, this method is reliable, reproducible, and less labor-intensive than chemical and enzyme-based protocol. All the procedure can be carried out in a single tube, reducing the risk for laboratory-induced contamination. The expense for Chelex^®^ method is negligible as compared to the one of commercial methods based on silica columns or magnetic beads costing from 2 to 6$ per test, which makes it particularly advantageous for future developments in low-income countries.

We also explored the clinical performances of our in-house *IS6110* PCR using the Chelex^®^ DNA extraction method in comparison to microscopy, culture and Xpert test. Results of the *IS6110* PCR appeared well correlated to smear microscopic semi-quantitative results (stratified from - to +++). The minimum concentration of *M. tuberculosis* DNA in microscopy-positive samples was estimated at 4 log_10_ genome equivalent/ml, in agreement with the minimal bacilli concentration request for smear microscopy (Palomino, 2005). Importantly, two third of the sputum smear-negative/culture positive tested positive for MTB DNA using the *IS6110* PCR. Previous studies reported a clinical sensitivity ranged from 91 to 97% in AFB positive-specimens and between 40 and 76% in AFB negative-specimens but specificity was ranging from 77 to 100% in both groups (Broccolo et al., 2003; El Khéchine et al., 2009; Lira et al., 2013). An *IS6110* assay is available on the m2000 Abbott system. Study by Tam et al. (2017) has recently reported high clinical performances in smear negative sputum using this assay. Other *IS6110* assays for use on open polyvalent PCR platform are also available (Bhembe et al., 2014; Obasanya et al., 2017). Our results suggest that using an optimized lysis and extraction method these *IS6110* in house and commercial assays may constitute a valuable option for routine molecular diagnosis. These tests contribute also to increase diversity and price competition between suppliers, and access to TB molecular diagnosis in resource-poor settings.

Sputum specimens containing AFB concentrations seated around the LOD were used to compare sensitivity of the *IS6110* PCR with the Xpert test. We focused the analysis on low bacterial load specimens because most of the demands from clinicians concern this type of specimen, which represent challenge for TB control, as a result of the difficulty of detecting smear-negative TB. Our results suggest that the combination of *IS6110* PCR and Chelex^®^ method exhibits a higher sensitivity to detect low sputum concentration of bacilli compared to Xpert test. The low sensitivity of Xpert for smear-negative specimens was previously described by Armand et al. (2011), but discordant with Miller et al. (2011). Notably, the new version of the MTB PCR cartridge (Xpert MTB/RIF Ultra), recently launched by Cepheid has included *IS6110* and *IS1081* targets to improve the sensitivity of the assay in sputum smear-negative samples (Chakravorty et al., 2017). The number sputum smear negative/culture positive sample tested in our study is one of the limit of the study. Low bacterial load specimens, obtained after serial dilutions were used for the comparison between the *IS6110* PCR and Xpert MTB/Rif assay. Previous studies have used diluted clinical samples to control the performance of molecular tests (Noordhoek et al., 2004; Akkerman et al., 2013).

Our results indicate that the Chelex^®^ method is highly effective for TB lysis and DNA enrichment in sputum. *IS6110* PCR combined with optimized lysis-extraction method achieved a level of analytical performances at least equivalent to these of the widely used Xpert kit on frozen sputum samples. Highly sensitive NATs are of great interest in TB diagnosis and are requested to reach an acceptable rate of MTB detection in subjects with paucibacillary specimens, a situation frequently encountered in patients with HIV and also in pediatric TB. This method should be considered as a possible alternative to fully automatized kits for TB DNA testing on open polyvalent PCR platform in central laboratories because of low cost and high throughput potential.

## Author Contributions

PK-D and SC-K conceived and designed the experiments. SG and LK contributed reagents and materials. PK-D and ET performed the experiments and wrote the draft. AB, PP, and ET revised the paper.

## Conflict of Interest Statement

The authors declare that the research was conducted in the absence of any commercial or financial relationships that could be construed as a potential conflict of interest.
